# Quantum Chemistry Insight into the Interactions Between Deep Eutectic Solvents and SO_2_

**DOI:** 10.3390/molecules24162963

**Published:** 2019-08-15

**Authors:** Mert Atilhan, Tausif Altamash, Santiago Aparicio

**Affiliations:** 1 Department of Chemical Engineering, Texas A&M University at Qatar, Doha 23874, Qatar; 2 Gas and Fuels Research Center, Texas A&M University, College Station, TX 77843, USA; 3 Qatar Environment and Energy Research Institute, Hamad Bin Khalifa University, Doha 23874, Qatar; 4 Department of Chemistry, University of Burgos, 09001 Burgos, Spain

**Keywords:** deep eutectic solvents, density functional theory, gas capture, SO_2_

## Abstract

A systematic research work on the rational design of task specific Deep Eutectic Solvents (DES) has been carried out via density functional theory (DFT) in order to increase knowledge on the key interaction parameters related to efficient SO_2_ capture by DES at a molecular level. A total of 11 different DES structures, for which high SO_2_ affinity and solubility is expected, have been selected in this work. SO_2_ interactions in selected DES were investigated in detail through DFT simulations and this work has generated a valuable set of information about required factors at the molecular level to provide high SO_2_ solubility in DES, which is crucial for enhancing the current efficiency of the SO_2_ capture process and replacing the current state of the art with environmentally friendly solvents and eventually implementing these materials in the chemical industry. Results that were obtained from DFT calculations were used to deduce the details of the type and the intensity of the interaction between DES and SO_2_ molecules at various interaction sites as well as to quantify short-range interactions by using various methods such as quantum theory of atoms in a molecule (QTAIM), electrostatic potentials (ESP) and reduced density gradients (RDG). Systematic research on the molecular interaction characterization between DES structures and SO_2_ molecule increases our knowledge on the rational design of task-specific DES.

## 1. Introduction

Sustaining air quality at desirable levels requires the constant management and control of greenhouse gases [1]. These gases have a severe impact on the environment and bring about its own ecological problems as industries continue to emit toxic greenhouse gases, which have reached unprecedented levels lately [2]. Industrial wastes in the form of flue gases are disposed mainly in the nitrogen (N_2_) and water vapor carrier stream with a low percentage concentration of carbon dioxide (CO_2_), 500 to 2000 ppm of sulfur dioxide (SO_2_) and a few ppm of nitrogen dioxide (NO_2_) [3,4]. Among the greenhouse gases (GHG) SO_2_ is not included among the direct ones (CO_2_, methane (CH_4_), nitrous oxide (N_2_O), hydrofluorocarbons (HFCs), perfluorocarbons, (PFCs), sulfur hexafluoride (SF_6_), nitrogen trifluoride (NF_3_)) and it is rather considered an indirect GHG. SO_2_ contributes to aerosol formation that can either absorb solar radiation on dark particles and cause warming, or forms cloud droplets, reflects radiation and cause cooling in the atmosphere. Direct GHGs have long atmospheric lifetimes and they have a high cumulative radiative-forcing effect. On the contrary, as an indirect GHG, SO_2_ has a short atmospheric lifetime and a lack of radiative-forcing effect, thus it has a relatively lower global warming effect over time. Despite being an indirect GHG and having a low concentration, SO_2_ could be extremely dangerous and hazardous to the environment as well as human health when released to atmosphere. Furthermore, due to its poisonous nature, it causes severe deterioration on most of the commonly used CO_2_ capture sorbents [3]. There are different scenarios for tackling these emissions and available state of the art technologies are employed depending on the needs of the processes. Therefore, both academia and industry are on the lookout for ways to improve the current state of the art on SO_2_ management and identifying an effective solvent for complying with the stringent rules for this industrial greenhouse gas.

SO_2_ is released to the atmosphere as a result of the combustion of fossil based energy sources [5]. Since SO_2_ is hazardous to human life and harmful to environment, controlling its concentration at its emission source as well as the reducing its amount in the atmosphere is crucially important [6,7]. In the case of its uncontrolled release to atmosphere, adverse effects such as acid rain may occur [8,9]. Furthermore, control of the SO_2_ emissions is also essential for the strategy of the sustainable environmental development of the human habitat. On the other hand, SO_2_ is an important reagent for several intermediates in chemical synthesis and production [10]. Nonetheless, capture of SO_2_ should not conflict with the sustainable development and must be economically feasible.

The conventional method of dealing with SO_2_ emissions includes amine scrubbing [11], ammonia scrubbing [12] and limestone scrubbing [13]. However, both these processes have well-known drawbacks such as solvent loss, corrosion for amine process and vast amount of produced wastewater along with huge quantities of CaSO_4_ as a by-product [14]. In recent years, more advanced adsorbents, or so-called sensing materials devices based on metal oxides (e.g. SnO_2_, WO_3_ and TiO_2_), have been proposed considering their selectivity, response time and recovery time [15,16,17] Nevertheless, due to the high temperature requirements of the processes that involve proposed semiconductors for SO_2_ raised concerns on their high power consumption requirement for such processes [18,19]. Thus, tackling the SO_2_ capture problem at low-to-moderate temperature conditions is still one of the major concerns and if achieved it will be a major milestone for deploying effective replacements to currently used materials for capturing SO_2_ [20,21,22]. For this purpose, during the past decade, ionic liquids (IL) have been considered effective greenhouse gas capture solvents due to their properties such as good thermal and chemical stability, non-flammability and almost null vapor pressure [23,24,25,26,27,28]. However, despite gravimetric capacity of studied IL yield compelling results, their high cost, cumbersome synthesis process, problematic purification and indeterminate toxicity did not make IL as an emerging new solvents for SO_2_ capture process until now [14].

Deep eutectic solvents (DES) are prepared typically (but not limited to) by mixing a quaternary ammonium halide salt considered as hydrogen bond acceptor (HBA), with hydrogen bond donor (HBD) molecule, which yields a structure with the halide and low melting point [29,30]. DES have recently been reflected as alternatives to IL that maintain most of their advantages (e.g. task-purpose tenability) and on the top of that avoid some of the major issues of IL such as economic and environmental concerns [31,32,33,34,35,36]. The synthesis of DES via low-cost sources including natural HBA and HBD, predictable and almost null toxicity and total biodegradability have made both industry and academia consider DES as alternative solvents for gas capture and separation processes in within the green chemistry framework. Having said that, in recent years there has been increasing interest in the utilization of DES for gas capture especially on CO_2_ capture and separation [37,38,39,40,41] however there are only a handful of studies that deal with the nature of the interaction between DES and SO_2_ [14,20,21,22,42,43,44,45,46,47,48,49,50]. Most of the available few studies are in experimental basis and simply deal with determination of SO_2_ solubility at low pressures in select DES systems [51]. Thus, there is a need on systematic approach to identify nanoscopic behavior on how SO_2_ interacts with DES, how the selection of HBA and HBD affect the interaction type and strength and draw conclusions on how an effective DES based SO_2_ capture solvent can be obtained. For this purpose, we have selected 11 different DESs and studied their interaction with SO_2_ by using Density Functional Theory (DFT). The content of this paper includes structure optimization and geometry characterization of the DES, determination of the energetic properties when DES and SO_2_ are interacting, detailed quantum based analysis such as quantum theory of atom in a molecule (QTAIM), electrostatic potential (ESP), reduced density gradient (RDG) and topological analysis of the studied systems. To the best of our knowledge, this paper is one of a kind that deals with numerous DES structures and their interaction with SO_2_ interactions in a systematic approach. We believe that this study will shed light on why and how DES can be used for SO_2_ capture process and assist others to design new purpose made DES for the same reason.

## 2. Methodology

In this work, a total of 11 different DESs were studied for their interactions with SO_2_. Candidates for SO_2_ capture solvents were determined after screening the available literature and shortlisting the most potential candidates for this task. After going through the literature [14,43,51,52,53], choline chloride (ChCl), 1-Butyl-3-methylimidazolium chloride (Bmim) and 1-Ethyl-3-methylimidazolium chloride (Emim) were selected as HBA and acetamide (Ac), citric acid (Ca), ethylene glycol (Eg), fructose (Ft), glycerol (Gy), lactic acid (La), levulinic acid (Lv), malic acid (Ma), phenylacetic acid (Pa) were selected as HBD. The selection of HBA was done based on the established know-how in literature as well as based on our previous studies. Furthermore, HBD selection was done considering simple representatives of amides, organic acids, polyols and sugar acting as HBDs, its low cost and its biocompatibility that fulfills the requirements for DESs development.

**Figure 1:**
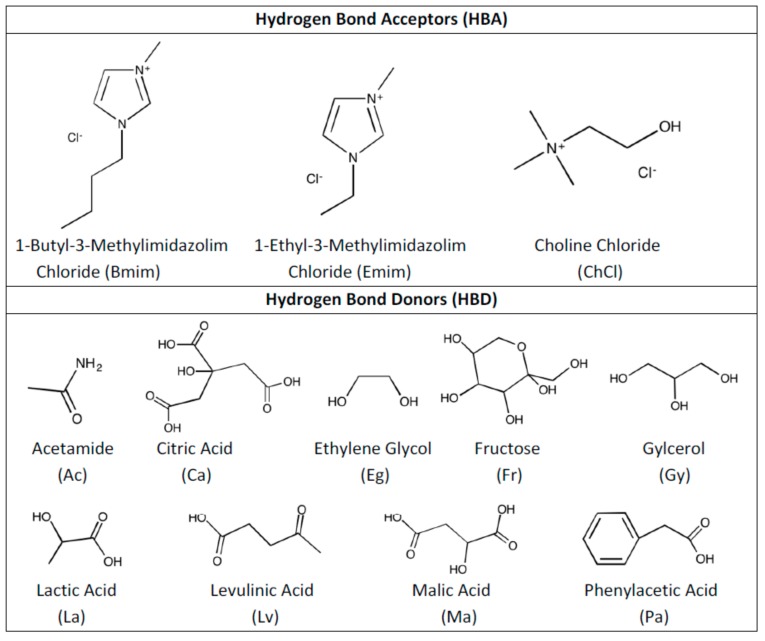
Molecular structures of hydrogen bond acceptor (HBA) and hydrogen bond donor (HBD) that are used in this work.

By using listed HBA and HBD, the following combinations were studied as DESs: Bmim:Ac, ChCl:Ca, ChCl:Eg, ChCl:Ft, ChCl:Gy, ChCl:La, ChCl:Lv, ChCl:Ma, ChCl:Pa, Emim:Ac and Emim:Eg. Figure 1 shows the molecular structures of HBA and HBD that are used in this work.

The initial structures for these DESs were built via Avogadro software [54] and DFT calculations were carried out via ORCA code [55]. B3LYP functional [56,57], together with Grimme’s DFT-D3 method [58], were chosen for considering dispersion interactions and the 6-311++G** basis set (i.e. B3LYP/6- 311++G** theoretical level) was selected for the DFT calculations. The total energy, ΔE, for each structure was calculated as the difference between the energy for the total cluster and the sum of the energies of the corresponding monomers, with the Basis Set Superposition Error (BSSE) corrected using the counterpoise procedure [59,60] and that is calculated in Equation (1) as:

(1) $$\Delta E_{tot}^{cp}=E_{sup}-\overset{n}{\underset{i=1}{\sum }}E_{m_{opt}}^{i}+\overset{n}{\underset{i=1}{\sum }}\left(E_{m_{f}}^{i}-E_{m_{f}}^{i*}\right)$$

$\Delta E_{tot}^{cp}\;$ is the counterpoise corrected superstructure; $E_{sup}\;$ is the optimized superstructure; $E_{m_{opt}}^{i}$ is the optimized single structure; $E_{m_{f}}^{i}$ is the optimized single structure while the coordinates are fixed at the original superstructure; $E_{m_{f}}^{i*}$ is the optimized single structure while the coordinates are fixed at the original superstructure and the other structure(s) are considered in the ghost orbital state.

Equation (1) is the general form of the BSSE corrected total energy for n component systems; however, in this study the studied interaction is between the SO_2_ molecule and DES superstructure, thus the above calculation over n is reduced down only for SO_2_. Binding energies were also estimated by considering the DES decomposed with its HBA and HBD components and Equation (2) was used for this purpose.

(2) $$E_{bind}=E_{DES}-\left(E_{HBA}+E_{HBD}+E_{SO2}\right)$$

Table S1 includes the values for *E_HBA_*, *E_HBD_* and *E_SO_2__*. Table S2 presents the calculated values for the parameters that are given in Equation (2). Real space functions and the characterization of the type of the inferred interactions were analyzed by using quantum theory of atoms in molecules (QTAIM), according to Bader’s AIM theory [61], by using the Multiwfn program code [62]. Intermolecular interactions between the studied DES and SO_2_ structures were characterized by the formation of bond critical points (BCPs) and corresponding electron density (*ρ*) as well as the Laplacian of the electron density (∇^2^*ρ*) values. BCPs of the charge density with inertia (3, −1) are located between two atoms (located at the interaction sites) and are calculated by QTAIM analysis, in which first the first derivative of the electron density reduces to zero from that calculated. Determination of the exact location of the BCPs and quantification of the *ρ* and ∇^2^*ρ* allow us to infer the type and the strength of the interaction between the interacting site atoms [63,64] and these values make quantitative formalisms possible [61,65]. The details of how BCPs are calculated were previously discussed elsewhere [66]. Reduced density gradient (RDG) analysis was used for obtaining the gradient-corrected functional of increasing quality [67,68,69]. RDG values were associated with isosurfaces by Johnson et al. [70] and they were associated with the non-covalent interactions of the studied superstructure. The RDG approach was used to discuss the nature and strength of the interactions displayed in the isosurface for the studied DES and SO_2_ superstructures. Furthermore, in order to decipher the nature of the charge transfer process between the various components of the studied DES and SO_2_ cluster, the density of states (DOS) analysis was carried out for investigating the orbital contributions from the various studied components. In addition to DOS analysis, as a complimentary analysis, the Homo–Lumo energies are studied as they are considered quantum mechanical indicators for determining chemical interactions since they provide an insight into the reactivity of the structures and the active site can be demonstrated by the distribution of the frontier orbital. The Homo–Lumo frontier orbital compositions for the studied structures were obtained at the same theory level of the DFT calculations and were demonstrated by using Avogadro software. Electrostatic potential (ESP) surface was studied in order to provide a visualization of total charge distribution of the DES+SO_2_ clusters and their corresponding relative polarity. Other than these analysis tools, some other discussions were carried out regarding the SO_2_ angle evolution and BCP distance evolution throughout the DFT calculations.

## 3. Results and Discussion

Each HBA and HBD was optimized as a first step of the DFT calculations. After optimizing each HBD and HBA (as well as SO_2_) structure, prior to the DES+SO_2_ DFT simulations, proposed DES structures were studied and optimized. For this purpose, various different initial geometries for Bmim:Ac, ChCl:Ca, ChCl:Eg, ChCl:Ft, ChCl:Gy, ChCl:La, ChCl:Lv, ChCl:Ma, ChCl:Pa, Emim:Ac and Emim:Eg were examined and for each of these possible configurations geometry optimization runs were performed at the above mentioned theory level. Most stable configurations for HBA and HBD were acquired from the PubChem^®^ database [71] and were optimized with the theory level that is mentioned in the previous section. This theory level has been selected based on our recent studies [72,73], which was proven for such DES and natural-DES (NADES) systems. The configurations for the HBA-HBD structure are based on the most stable configuration of the studies systems. Various alternative configurations have been considered during the optimization of the DES structures. Final structures that are considered for DES were determined based on the analysis of the H-bonding and its length since it plays important role on the formation of the DES structures. Furthermore, the energetic analyses of the DFT geometry optimizations were considered for the selection of the most stable configuration of the studied structures. It was observed that the structure of the HBA stayed unchanged, whereas the structure of the HBD changes dramatically during the geometry optimization of the studied DES systems. For each studied DES structure, 2 different configurations were studied (except for ChCl:Ca for which 3 different configurations used) depending on the H-bond formation alternatives between the HBA and HBD. Among these studied configurations, the lowest energy yielding cases were selected as the final optimized DES structure and they were used for the further simulations with SO_2_ interactions. 

While dealing with the geometry optimization of these initial structures in our previous publications, an iterative method was used to figure out the most suitable theory level that yields reasonable structures. Until we reached the basis set and the theory level that is used in this work, several other simpler theories were used, for which the risk of converging to a local minima is highly likely. However, the mentioned theory is complex enough to avoid local minima and which was also confirmed with the non-imaginary frequencies.

**Figure 2:**
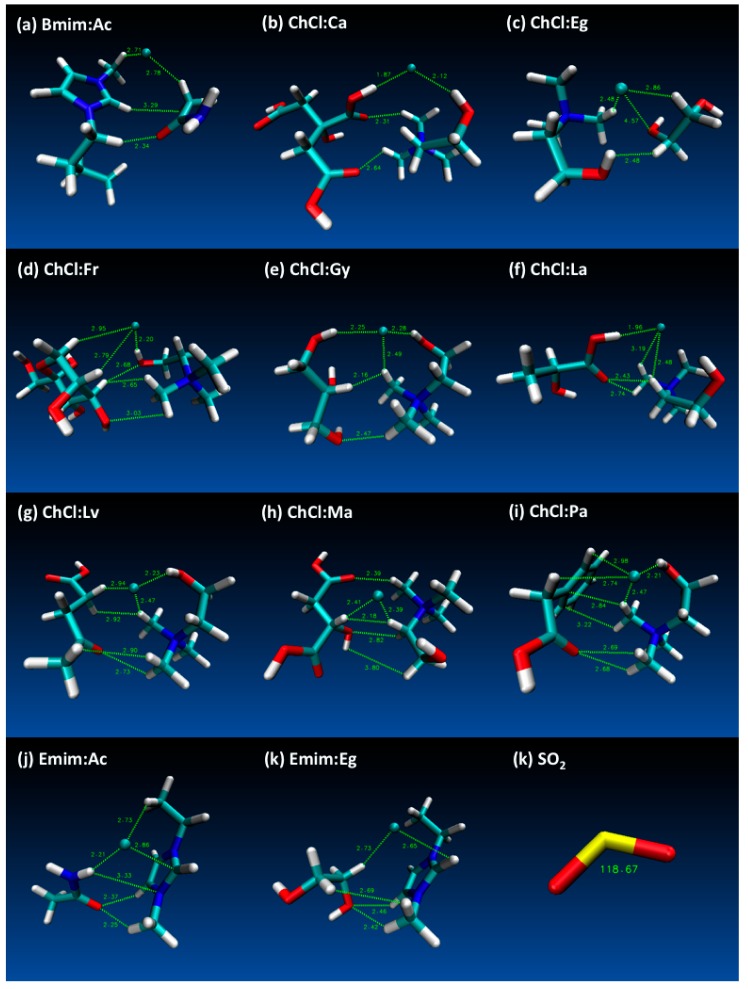
Geometry optimized deep eutectic solvent (DES) structures (and SO_2_) that are studied in this work (displayed distances are in Å and SO_2_ angle is degrees °).

The final energies for each of the studied DES structures are provided in Table S3 (Electronic Supporting Information—ESI) and according to these results lowest energy yielding DES cases have been selected for further simulations with SO_2_. The final optimized structures for DES and SO_2_ are provided in Figure 2, which also displays notable distances between HBD and HBA structures obtained at the end of the geometry optimizations. Most notable distances were highlighted for Cl^−^⋅⋅⋅H, Cl^−^⋅⋅⋅O, C⋅⋅⋅H, C⋅⋅⋅C, O⋅⋅⋅H, O⋅⋅⋅C and H⋅⋅⋅H interactions in Figure 1 and the distances that are displayed on figures Figure 2a–k shows the integrity of the DES structures were attained at reasonable interatomic distances between the HBA (for both cation and anion) and HBD molecules. Furthermore, Figure 1k shows the SO_2_ angle as 118.67°, which confirms the literature values [74].

Since SO_2_ is a relatively small molecule, that allows placement of SO_2_ molecule around the DES structure in various different spatial positions. For this purpose, 4 different spatial positions were determined and studied for each DES for the placement of SO_2_ molecule. While identifying these positions, cation and anion for HBA and HBD were considered for their potential H-bonding sites that can form stronger interactions with O and as per the possible interactions within the van der Waals radius [75]. Then, based on this consideration geometry optimizations for these different positions were carried out at mentioned theory level. Once important BSSE counterpoise correction procedure was applied, the lowest energy yielding DES+SO_2_ structures were identified for detailed quantum chemical analysis. Table S2 shows the final optimized interaction energies for the studied DES+SO_2_ systems and the details of the BSSE counterpoise correction results. Figure S1 (Supporting Information) shows the DES+SO_2_ systems that was studied and Table S3 (Supporting Information) indicates the interaction sites for these systems. 

At the end of the DFT simulations at the mentioned theory level, the evolution of the path length throughout the simulation for the most notable BCP (explained in detail in the following sections) that occurs between the DES and the SO_2_ molecule has been examined. As per Figure S2, the BCP path length for all of the systems has eventually been lowered and finally stabilized at certain values at the end of the simulation steps, confirming the successful convergence of the structures. Furthermore, as a primary indication of potential structural disruption on SO_2_ molecule that might lead to bond breaking and reactive cases, the bond angle between S and O atoms for SO_2_ has been analyzed throughout the simulation steps. As per Figure S3, minor changes in SO_2_ bond angle has been recorded and reduction in the bond angle was limited to around ~3°, which confirms the structural integrity of the SO_2_ molecule despite its interaction with the DES structures.

As a first approximation, SO_2_ capture at the molecular level were approximated with the strength of the interactions between DES and SO_2_ molecule were estimated as shown in Equation (2) with the consideration of DES is composed of HBA and HBD and are interacting with SO_2_ molecule. According to the reported values in Table S2, ChCl:Ca+SO_2_ has the highest and ChCl: Eg+SO_2_ ≈ Emim:Eg+SO_2_ has the lowest binding energies. However, when the binding energies are observed as a pivotal reference for comparison purposes for the studied structures, it is observed that the variation of the binding energy is not dramatic (average *E_bind_* ≈ −0.03 eH with *σ(stdev)* = 0.007). Through the comparison of between the HBA and HBD relations with SO_2_ and the binding energy values, one could obtain further characterization of either of the HBA or HBD is dominant in SO_2_ affinity. In most of the cases, SO_2_ capture would be mainly due to the HBA and the cation part of the IL that is considered for HBA, except for couple of cases. Furthermore, in the case of HBA⋅⋅⋅SO_2_ affinity, we observed that the interactions are mostly localized between the O atom of the SO_2_ and the cation part of the IL. In contrary with this, when HBD dominates the SO_2_ affinity, O atom in the HBD interacts with the S atom in the SO_2_ molecule.

**Figure 3:**
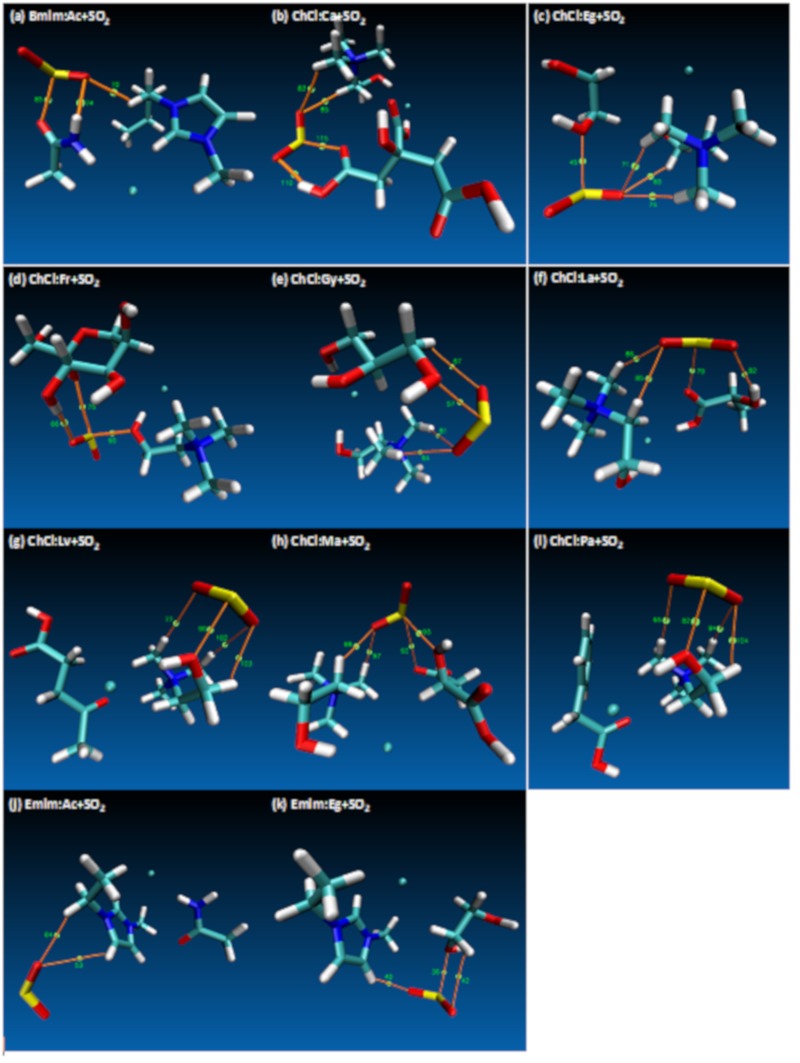
Atom in Molecule (AIM) analysis of interactions and bond critical points (BCP) involving DES+SO_2_ systems. Electron density (ρ) and Laplacian of electron density (∇^2^ρ) for displayed BCPs are reported in Table 1.

In order to evaluate why the interaction energies were ranked as such, a more detailed QTAIM analysis were carried out. Table 1 shows the *ρ* and ∇^2^*ρ* values for the calculated BCPs calculated between the interacting site atoms as a guide to study the nature of bonding in molecular systems and Figure 3 displays the visual representation of the BCPs for the studied DES+SO_2_ systems. The *ρ* at the BCP is a direct measure of how strongly the interaction site atoms are binding with each other [76,77]. According to this, *ρ* for ChCl:Ca+SO_2_ (BCP 83 occur between O⋅⋅⋅H) is the highest observed value, which is in line with the highest interaction or binding energy that was mentioned previously.

When the BCPs are analyzed visually from Figure 3a–k, for all of the cases the SO_2_ molecule interacts with both HBA and HBD except for ChCl:Lv+SO_2_, ChCl:Pa+SO_2_ and Emim:Ac+SO_2_. Among these ChCl:Lv+SO_2_ and Emim:Ac+SO_2_ has yielded low interaction energies and low *ρ*, whereas for the ChCl:Pa+SO_2_ case has quite a high affinity between the S⋅⋅⋅O sites; which confirms the Lewis electron pair model based high interaction [78] that is reported in Table S2 for ChCl:Pa+SO_2_.

The reported ∇^2^*ρ* values in Table 1 delivers physical foundation for the electron pair model of Lewis [79,80] and it can be joint with other concepts in electronic structure theory of molecules. They are mostly reported as positive values (∇^2^*ρ* > 0), which indicate that the interaction is subjugated by the contraction of *ρ* towards each nucleus and for such cases the net forces of repulsion act on the nuclei. On the other hand, there are some negative values (∇^2^*ρ* < 0) appeared in Table 1, which indicates the concentration of charge towards the interaction line, accumulation of the electron density in the region between the two bonded atoms and thus creating an attractive force and a bound shared interaction. As the local total energy density at observed BCP continues to reach more negative then ∇^2^*ρ* shall become progressively more positive in value as *ρ* increases. This result is at odds with the assertion that ∇^2^*ρ* should decrease and reach to negative values as local total energy density at observed BCP becomes progressively more negative for shared bonded interactions. 

**Figure 4:**
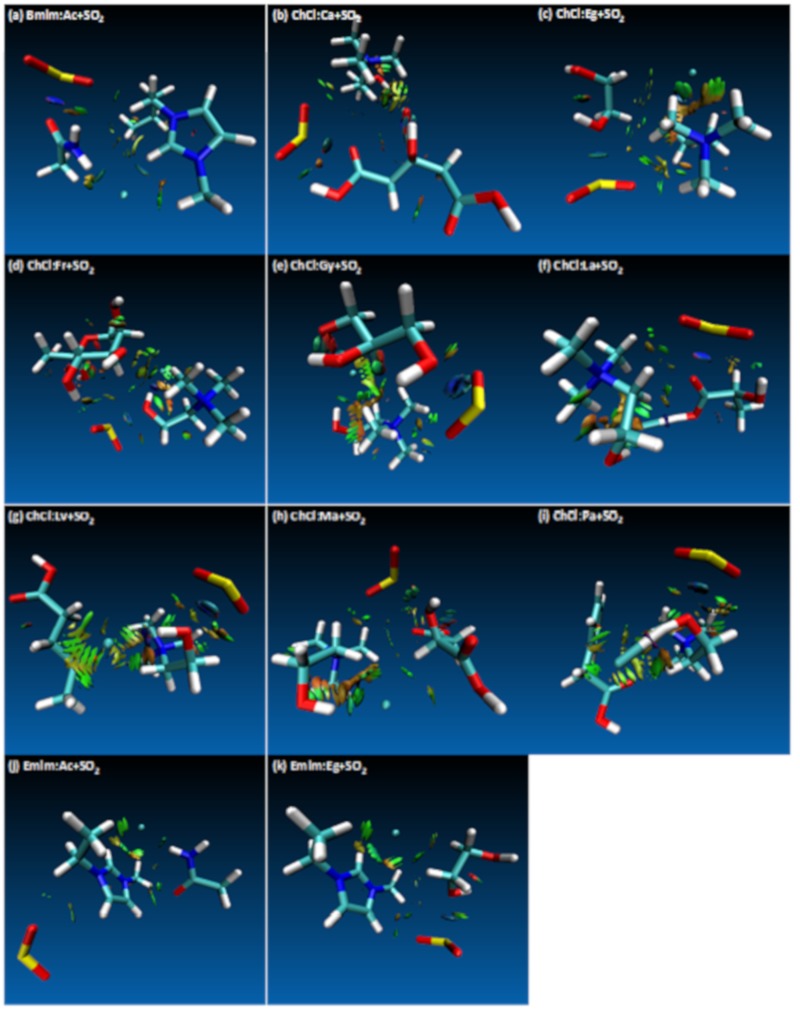
Reduced Density Gradient (RDG) iso-surfaces of the studied systems. van der Waals type of attraction are displayed with green isosurfaces, strong repulsion cases are displayed with red isosurfaces and for strong attractions are displayed with blue isosurfaces.

The effect of different HBAs can be analyzed since there are 3 different HBAs used among the studied DES systems. As mentioned above, the highest interaction energy case was observed for ChCl:Ca+SO_2_ system and it was confirmed by the value of the *ρ.* On the other hand, when other cations were considered for different HBAs, the following interaction strength has been observed (Table 1) Bmim:Ac+SO_2_ > Emim:Eg+SO_2_ > Emim:Ac+SO_2_, which is in line with the interaction energies shared in Table S2. Among the cations, ChCl has the highest SO_2_ affinity effect when compared to Bmim and Emim. According to Table 1 and Figure 2, in most of the studied cases cation/HBA dominates the interaction between the O atom of the SO_2_. There are only 3 cases in which HBD has the leading role on the SO_2_ interaction; these are ChCl:Eg+SO_2_, ChCl:Ma+SO_2_ and ChCl:Fr+SO_2_ cases. As per Figure 2, for ChCl:Eg+SO_2_, interaction takes place between O(Eg/HBD)⋅⋅⋅S(SO_2_), for the case of ChCl:Ma+SO_2_ interaction takes place between O(Ma/HBD)⋅⋅⋅S(SO_2_) and for the case of ChCl:Fr+SO_2_ interaction takes place between H(Fr/HBD)⋅⋅⋅O(SO_2_) sites. On the other hand, for the rest of the cases for which HBA has the dominating role, H(cation/HBA)⋅⋅⋅O(SO_2_) interaction has been observed. It can be concluded that cations of the HBA are the main responsible structure for the SO_2_ affinity, for which there are few exceptions with HBD takes care of this role. However, all of the interactions of both HBA and HBD with respect to SO_2_ has been visualized with RDG contour plots. The strength and the type of the interactions of the HBA/HBD can be visualized with the RDG plots that are presented in Figure 4. According to Figure 4, most of the molecular interactions fall within van der Waals type of attraction (green isosurfaces). Small *ρ* and ∇^2^*ρ* values leads to chemical bonding for weak interactions, large values lead to strong repulsion cases (red isosurfaces) and for strong attractions are displayed with blue isosurfaces. When Figure 4 is studied in detail, the effect of SO_2_ did not have disrupting effect between the HBA and HBD, which are evident from the available weak interaction isosurfaces, thus confirming the integrity of the DES systems.

**Figure 5:**
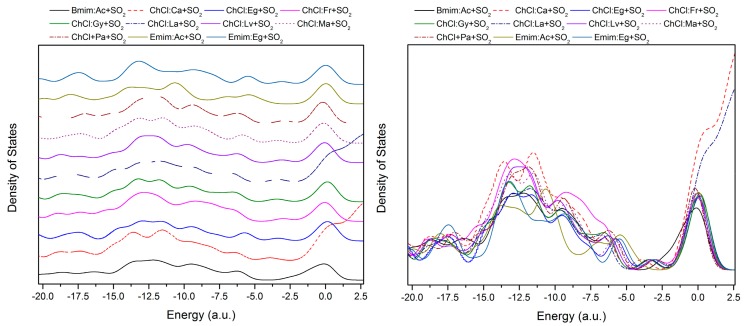
Density of states (DOS) as a function of orbital energy, E, for studied DES+SO_2_ structures calculated at B3LYP/6-311++g(d,p) level. (**left**—combined DOS plot for all DES+SO_2_ systems; **right**—stacked with constant y-offset value on the DOS plot to separate the curves).

Once plotted, the density-of-states (DOS) graph can provide insight into the number of states in the unit energy interval and can be used to evaluate the nature of the electron structure. Furthermore, DOS analysis can also be used to decipher the nature of the charge transfer that occurs between the various active sites of the DES and SO_2_ molecules. The DOS plot is provided in Figure 5 for all the studied combined DES+SO_2_ structures. Homo and Lumo separations are clearly observed approximately between a −5.0 a.u. and −2.0 a.u. range. One of the most important observations that can be made from the DOS plot is the broad nature of the calculated DOS peaks, which corresponds to relatively weaker interactions between the DES⋅⋅⋅SO_2_ structures and this conclusion is in line with the previously confirmed van der Waals interactions between DES⋅⋅⋅SO_2_ in QTAIM analysis. It is observed that the DOS feature for ChCl:Ca+SO_2_ and ChCl:La+SO_2_ increasingly perturbed from Homo to Lumo. Moreover, for the same structures, the DOS features are considerably altered compared to the rest of the studied DES+SO_2_ structures, which indicate charge donations from orbitals that are much lower in energy compared to that of the HOMO. The DOS feature for Emim:Ac+SO_2_ within the range of −15.0 to −10.0 a.u. is reduced in intensity compared to the rest of the studied DES+SO_2_ structures. This could be the reason Emim^+^ plays a relatively less attractive role compared to Ch^+^ in the charge transfer process, as it was also evident from the interaction energies. 

**Figure 6:**
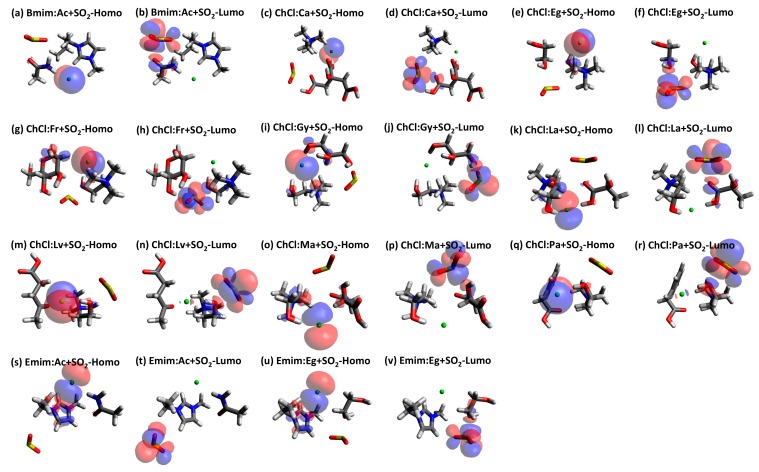
HOMO-LUMO graphs for each DES+SO_2_ structure (positive (blue) and negative (red) isosurfaces).

Since there is no significant shifting of the peak it can be concluded that the behavior of the studied DES+SO_2_ systems almost has similar behavior with minor differences on the intensities on the peaks. HOMO-LUMO plots for the studied DES+SO_2_ systems are provided in Figure 6 with isosurfaces indicated by blue (positive) and red (negative). From Figure 6, it can be seen that HOMO always formed around the Cl^−^ anion and LUMO formed around the SO_2_ molecule. The Homo–Lumo values and gaps that are presented in Table S4 (Supporting Information). Although Homo-LUMO gap information is not directly related to the solubility related properties, it is rather preferred to infer the stability and the reactivity of the studied structures. In this regard, one shall expect higher molecular stability and lower reactivity in chemical reactions for larger calculated HOMO–LUMO gap values. Having said that, Table S4 shows that these structures are highly stable. Furthermore, the presented values in Table S4 indicate that they are within the visible region, which refers to the light absorption in the UV region.

**Figure 7:**
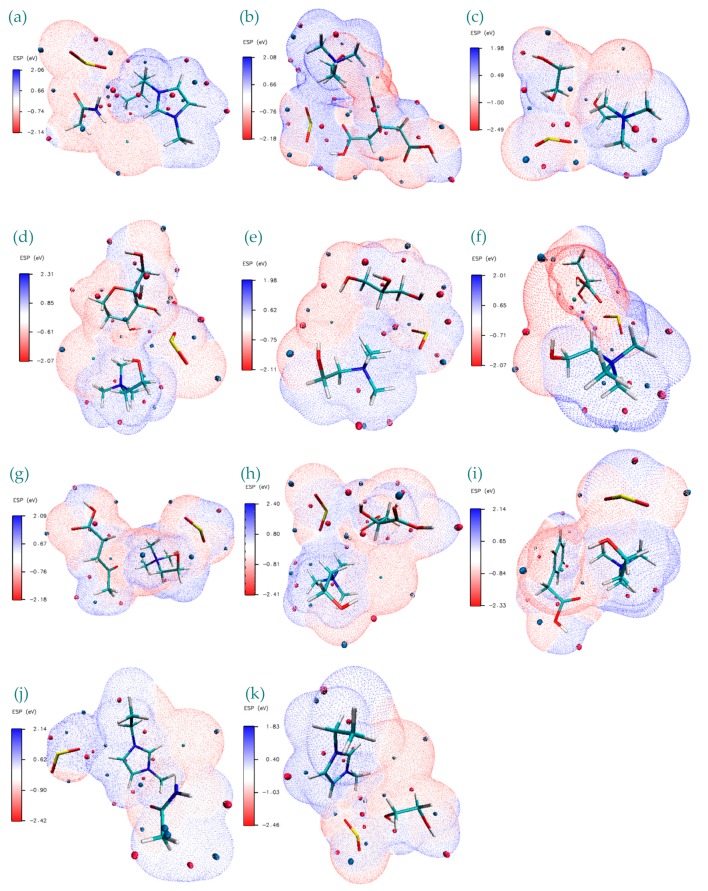
Electrostatic potential (ESP) surface map visualization for studied DES+SO_2_ structures. Blue, white and red correspond to ESP varying from min to max level, the blue and red spheres correspond to ESP surface minima and maxima, respectively. ESP ranges are included in the legend at each figure panel. (**a**) Bmim:Ac+SO_2_, (**b**) ChCl:Ca+SO_2_, (**c**) ChCl:Eg+SO_2_, (**d**) ChCl:Fr+SO_2_, (**e**)ChCl:Gy+SO_2_, (**f**) ChCl:La+SO_2_, (**g**) ChCl:Lv+SO_2_, (**h**)ChCl:Ma+SO_2_, (**i**) ChCl:Pa+SO_2_, (**j**) Emim:Ac+SO_2_, (**k**) Emim:Eg+SO_2_

ESP isosurface plots provide an effective visualization of total charge distribution and relative polarity of the studied DES+SO_2_ structures. These plots are provided in Figure 7a–k. Close inspection of these isosurfaces reveals that for the cases where ChCl is used, HBA area is covered with blue (−) isosurfaces corresponding to effect of the abundant π-electron cloud and aggregation of electron density; whereas for Bmim and Emim cases it is observed that red (+) isosurfaces are observed around the HBA vicinity corresponding repulsion of the proton by the atomic nuclei or nucleophilic reactivity.

ESP isosurface plots provide an effective visualization of total charge distribution and relative polarity of the studied DES+SO_2_ structures. These plots are provided in Figure 7a–k. Close inspection of these isosurfaces reveals that for the cases where ChCl is used, HBA area is covered with blue (−) isosurfaces corresponding to effect of the abundant π-electron cloud and aggregation of electron density; whereas for Bmim and Emim cases it is observed that red (+) isosurfaces are observed around the HBA vicinity corresponding repulsion of the proton by the atomic nuclei or nucleophilic reactivity. Regarding SO_2_, for all the cases, it is dominated by either blue minimum or red maximum, except for the case of ChCl:Ca+SO_2_, for which it has a well-balanced minimum and maximum isosurfaces surrounding the molecule. This corresponds to stronger bonding between the H⋅⋅⋅O. Furthermore; easy electron transfer from positive charges located on oxygen atoms from C-O group of Ca are happening between the additional negative regions around the SO_2_. As observed in most of the cases, negative charge of O atoms in SO_2_ stabilizes positive charge at the counterpart site of the DES structure. The narrow white border-like regions distinctly separate the extents of the blue regions to red regions and these are related to the near-neutral parts between negative/positive regions. ESP isosurfaces for the DES+SO_2_ structures presented in Figure 7 show that the greatest negative electrostatic potential is located mainly over the O=, N≡ from imino groups (>C=N–) and =S= in SO_2_ with a minimum value ranging from between −2.5 eV and −2.0 eV. On the other hand, positive region is localized mostly on the Cl^−^ anion of the HBA. From Figure 7 it can be inferred that positive charge is more spread over the C–H of the HBD and S=O. 

## 4. Conclusions

In this work, a thorough DFT analysis has been conducted on 9 different selected DES compounds on their affinity towards SO_2_, as they are being considered as an alternative solvents for industrial purposes. A detailed QTAIM has provided a mechanism of interaction sites and the strongest interaction paths between the DES and SO_2_. Quantification of the QTAIM analysis is explained in detail and is discussed in the manuscript. These results were in line with the calculated interaction energies. Furthermore, RDG analysis visually proved the interaction type between the studied structures and confirmed a dominant van der Waals type interaction between DES and SO_2_. DOS studies were also used to infer the nature of the charge transfer that occurs between the various active sites of the DES and SO_2_ molecules and these results were coupled with Homo-Lumo analysis as well. The DOS results helped us to identify which anion or cation from the corresponding HBA plays the major role on the charge transfer process. Additionally, time-consuming ESP analysis enabled the drawing of isosurfaces that provided an effective visualization on the total charge distribution and relative polarity of the studied DES+SO_2_ structures. With the aid of the ESP analysis, the positive and negative electrostatic potentials were identified and they were analyzed accordingly. 

Based on these studies we draw these conclusions:

(i) These results are quite useful in order to show the Lewis model interactions and acidity of DESs as absorbents that are considered for SO_2_ absorption. Furthermore, the chemical and kinetic stability of the DES structures was confirmed when they are in contact with SO_2_, which is a pre-indicator of these solvents for cyclic use and their regenerable nature.
(ii) Both QTAIM and structure analysis results show that strong interactions contribute to SO_2_ absorption, which are controlled mainly with the cation component of the HBA. These interactions are mostly localized between the O atom of the SO_2_ and the cation part of the IL. In contrast with this, when HBD dominates (just a couple of cases) the SO_2_ affinity, O atom in the HBD interacts with the S atom in the SO_2_ molecule.
(iii) Despite the calculation of overall total energies and more importantly binding energies not showing a wide range of variation, it is obvious that the selection of HBD makes the structure sensitive to the SO_2_ interaction.

This study is one of the very few systematic computational analyses on the utilization of DES considered for SO_2_ capture and it will open further discussions on this topic for not only SO_2_ management but also for other acid/sour gases such as N*_x_*O, SO*_x_* and H_2_S in due course. 

Thus, in light of the molecular interaction findings, qualitative trends on the absorption of SO_2_ with novel DES can be focused more on the investigation of HBA(cation)⋅⋅⋅SO_2_ systems. Systematic research on this would assist with building knowledge about those factors at the molecular level, allowing an approach to the rational design of task-specific DES or NADES for future applied studies. 

There might exist other possible configurations other than the complex interaction pathways for the interaction of DES (HBA and HBD) with SO_2_ within the proposed cluster model. In order to investigate all the available interaction configurations, there is a need for systematic molecular dynamics simulations that should be evaluated in light of the presented quantum chemical calculations, which is out of the scope of the current study. Obviously, information related to SO_2_ solubility in DES structures in the bulk phase would give more practical information regarding these solvents’ performance at near-real life process conditions. In order to do that, molecular dynamics simulations are required, which will be disseminated in due course to provide insights from the process point of view.

## Figures and Tables

**Table 1:** Bond critical point (BCP) results for studied structures (Electron density, *ρ* and Laplacian of electron density, ∇^2^*ρ* values are reported).

Structure	BCP No.	*ρ* × 10^3^/a.u.	∇^2^*ρ* × 10^2^/a.u.	Structure	BCP No.	*ρ* × 10^3^/a.u.	∇^2^*ρ* × 10^2^/a.u.
Bmim:Ac+SO_2_	70	1.24	3.11	ChCl:La+SO_2_	79	0.800	1.36
	74	1.14	3.81		82	−283	−99.0
	85	0.498	12.1		85	0.965	2.10
ChCl:Ca+SO_2_	83	2.06	6.81		86	0.842	2.67
	85	1.46	8.87	ChCl:Lv+SO_2_	77	1.07	3.02
	110	0.872	2.75		90	−322	99.9
	115	0.784	2.10		102	−279	−97.2
ChCl:Eg+SO_2_	45	1.31	8.91		103	0.866	2.74
	60	0.835	2.50	ChCl:Ma+SO_2_	92	0.829	1.90
	71	0.620	1.80		93	1.50	7.00
	74	1.10	2.99		97	1.31	3.65
ChCl:Fr+SO_2_	66	2.64	7.59		98	1.01	3.00
	75	0.889	2.26	ChCl:Pa+SO_2_	66	0.679	1.84
	90	1.07	8.95		82	1.09	10.9
ChCl:Gy+SO_2_	57	−276	−95.3		94	1.25	3.64
	64	1.11	2.67		104	0.908	3.12
	67	0.796	2.22	Emim:Eg+SO_2_	36	0.854	9.83
	81	−248	−50.1		40	1.07	2.57
Emim:Ac+SO_2_	53	0.637	1.20		42	0.660	1.13
	64	0.719	2.02				

## Supplementary Materials

The Supplementary Materials are available online. This section includes the initial energies of optimized DES, DES+SO_2_ systems that was studied and the interaction energies for these systems, Homo/Lumo energies and energy gaps, distance evolution between BCP forming sites (DES⋅⋅⋅SO_2_) and SO_2_ angle evolution for each DES+SO_2_ case throughout the geometry optimization.
