# Supplementary material for: Quantum Chemistry Insight into the Interactions Between Deep Eutectic Solvents and SO2
Source: Molecules. 2019 Aug 15;24(16):2963. doi: 10.3390/molecules24162963 (PMC6720665; doi:10.3390/molecules24162963)
Supplement: Supplementary file 1 [file molecules-24-02963-s001.pdf]

Quantum Chemistry Insight Into the Interactions Between Deep Eutectic Solvents and SO_2

Mert Atilhan *[1,2], Tausif Altamash [3] and Santiago Aparicio *[,4]

[1] Department of Chemical Engineering, Texas A&M University at Qatar, Doha 23874, Qatar.
[2] Gas and Fuels Research Center, Texas A&M University, College Station, TX 77843, USA
[3] Qatar Environment and Energy Research Institute, Hamad Bin Khalifa University, Doha 23874, Qatar
[4] Department of Chemistry, University of Burgos, Burgos 09001, Spain
* Correspondence: mert.atilhan@tamu.edu (M.A.); sapar@ubu.es (S.A.)

ELECTRONIC SUPPORTING INFORMATION

Table S1. DFT results for optimized initial structures for HBA, HBD and SO2 molecules.

	Structures	E (eH)
**HBA**	BmimCl	-883.3831217
	EmimCl	-804.7954558
	ChCl	-788.9625135
**HBD**	Acetamide	-209.1704337
	Citric Acid	-759.9425707
	Ethylene Glycol	-230.2091851
	Fructose	-687.0468922
	Glycerol	-344.7199347
	Lactic Acid	-343.5523307
	Levulinic Acid	-420.9460304
	Malic Acid	-532.0988742
	Phnylacetic Acid	-460.0018162
**Gas**	SO2	-548.5454487

Table S2. DFT results for optimized superstructure (sstr) , optimized single structure while the coordinates are fixed at original superstructure and the other structure is considered at ghost orbital, and counterpoise corrected superstructure.

Structure	E_{sstr} (eH)	$E\_m^{i}_{opt}$ (eH)		$E\_m^{i}_{f}$ (eH)		$E\_m^{i*}_{f}$ (eH)		E_{Bind} (eH)	E_{int_cp} (eH)
		DES	SO_2	DES	SO_2	DES	SO_2		
Bmim:Ac+SO_2	-1641.137	-1092.632	-548.545	-1092.628	-548.544	-1092.628	-548.547	-0.03754	0.038
Emim:Ac+SO_2	-1562.536	-1014.038	-548.545	-1014.037	-548.545	-1014.037	-548.547	-0.02472	0.046
Emim:Eg+SO_2	-1583.573	-1035.067	-548.545	-1035.059	-548.545	-1035.060	-548.548	-0.02282	0.036
ChCl:Ca+SO_2	-2097.494	-1549.022	-548.545	-1549.016	-548.545	-1549.017	-548.548	-0.04379	0.070
ChCh:Eg+SO_2	-1567.738	-1019.233	-548.545	-1019.230	-548.545	-1019.230	-548.548	-0.02081	0.037
ChCl:Gy+SO_2	-1682.262	-1133.765	-548.545	-1133.759	-548.545	-1133.759	-548.547	-0.03417	0.045
ChCl:La+SO_2	-1681.095	-1132.599	-548.545	-1132.587	-548.545	-1132.587	-548.548	-0.03465	0.045
ChCl:Lv+SO_2	-1758.483	-1209.991	-548.545	-1209.989	-548.545	-1209.990	-548.547	-0.02916	0.050
ChCl:Ma+SO_2	-1869.631	-1321.139	-548.545	-1321.136	-548.545	-1321.137	-548.548	-0.02393	0.051
ChCl:Pa+SO_2	-1797.538	-1249.058	-548.545	-1249.054	-548.545	-1249.055	-548.547	-0.02805	0.063
ChCl:Fr+SO_2	-2024.583	-1476.101	-548.545	-1476.107	-548.545	-1476.107	-548.548	-0.02806	0.060

Table S3. DES+SO_2(P1-P2-P3-P4) structure interaction energies

Structure	Energy	Interaction Site
BmAcSO2_p01	-1641.148568	
BmAcSO2_p02	-1641.144505	
BmAcSO2_p03	-1641.148068	
BmAcSO2_p04	-1641.13654	***H(Bmim/cation-HBA)---O(SO2)***
EmAcSO2_p01	-1562.558486	
EmAcSO2_p02	-1562.558489	
EmAcSO2_p03	-1562.536054	***H(Emim/cation-HBA)---O(SO2)***
EmAcSO2_p04	-1562.549736	
EmEgSO2_p01	-1583.588249	
EmEgSO2_p02	-1583.590161	
EmEgSO2_p03	-1583.588432	
EmEgSO2_p04	-1583.572911	***H(Emim/cation-HBA)---O(SO2)***
ChClCaSO2_p01	-2097.496827	
ChClCaSO2_p02	-2097.498376	
ChClCaSO2_p03	-2097.498917	
ChClCaSO2_p04	-2097.494318	***H(Ch/cation-HBA)---O(SO2)***
ChChEgSO2_p01	-1567.751228	
ChChEgSO2_p02	-1567.750135	
ChChEgSO2_p03	-1567.751219	
ChChEgSO2_p04	-1567.737960	***O(Eg/HBD)---S(SO2)***
ChClGySO2_p01	-1682.270703	
ChClGySO2_p02	-1682.270765	
ChClGySO2_p03	-1682.267707	
ChClGySO2_p04	-1682.262070	***H(Ch/cation-HBA)---O(SO2)***

Table S3. (con't) DES+SO_2(P1-P2-P3-P4) structure interaction energies

Structure	Energy	Interaction Site
ChClLaSO2_p01	-1681.107947	
ChClLaSO2_p02	-1681.103574	
ChClLaSO2_p03	-1681.103584	
ChClLaSO2_p04	-1681.094941	***H(Ch/cation-HBA)---O(SO2)***
ChClLvSO2_p01	-1758.494000	
ChClLvSO2_p02	-1758.491672	
ChClLvSO2_p03	-1758.491649	
ChClLvSO2_p04	-1758.483149	***H(Ch/cation-HBA)---O(SO2)***
ChClMaSO2_p01	-1869.650684	
ChClMaSO2_p02	-1869.645771	
ChClMaSO2_p03	-1869.645641	
ChClMaSO2_p04	-1869.630769	***O(Ma/HBD)---S(SO2)***
ChClPaSO2_p01	-1797.555106	
ChClPaSO2_p02	-1797.554438	
ChClPaSO2_p03	-1797.548155	
ChClPaSO2_p04	-1797.537826	***H(Ch/cation-HBA)---O(SO2)***
ChClFrSO2_p01	-2024.593546	
ChclFrSO2_p02	-2024.589858	
ChclFrSO2_p03	-2024.589092	
ChclFrSO2_p04	-2024.582912	***H(Fr/HBD)---O(SO2)***

Table S4. DFT results on HOMO (E_{HOMO}) and LUMO (E_{LUMO}) energies; HOMO-LUMO energy gap (ΔE_G).

**Structure**	**E_{HOMO} / eV**	**E_{LUMO} / eV**	**ΔE_G / eV**
Bmim:Ac+SO_2	-5.3880	-3.2998	2.088
Emim:Ac+SO_2	-5.4208	-4.4488	0.972
Emim:Eg+SO_2	-5.8618	-3.4798	2.382
ChCl:Ca+SO_2	-6.3718	-4.6476	1.724
ChCh:Eg+SO_2	-5.8618	-3.4798	2.382
ChCl:Gy+SO_2	-6.3437	-4.5654	1.778
ChCl:La+SO_2	-6.5786	-4.1019	2.476
ChCl:Lv+SO_2	-6.0367	-3.0935	2.943
ChCl:Ma+SO_2	-5.6702	-4.1450	1.525
ChCl:Pa+SO_2	-5.9989	-3.4808	2.518
ChCl:Fr+SO_2	-6.0857	-2.2375	3.848

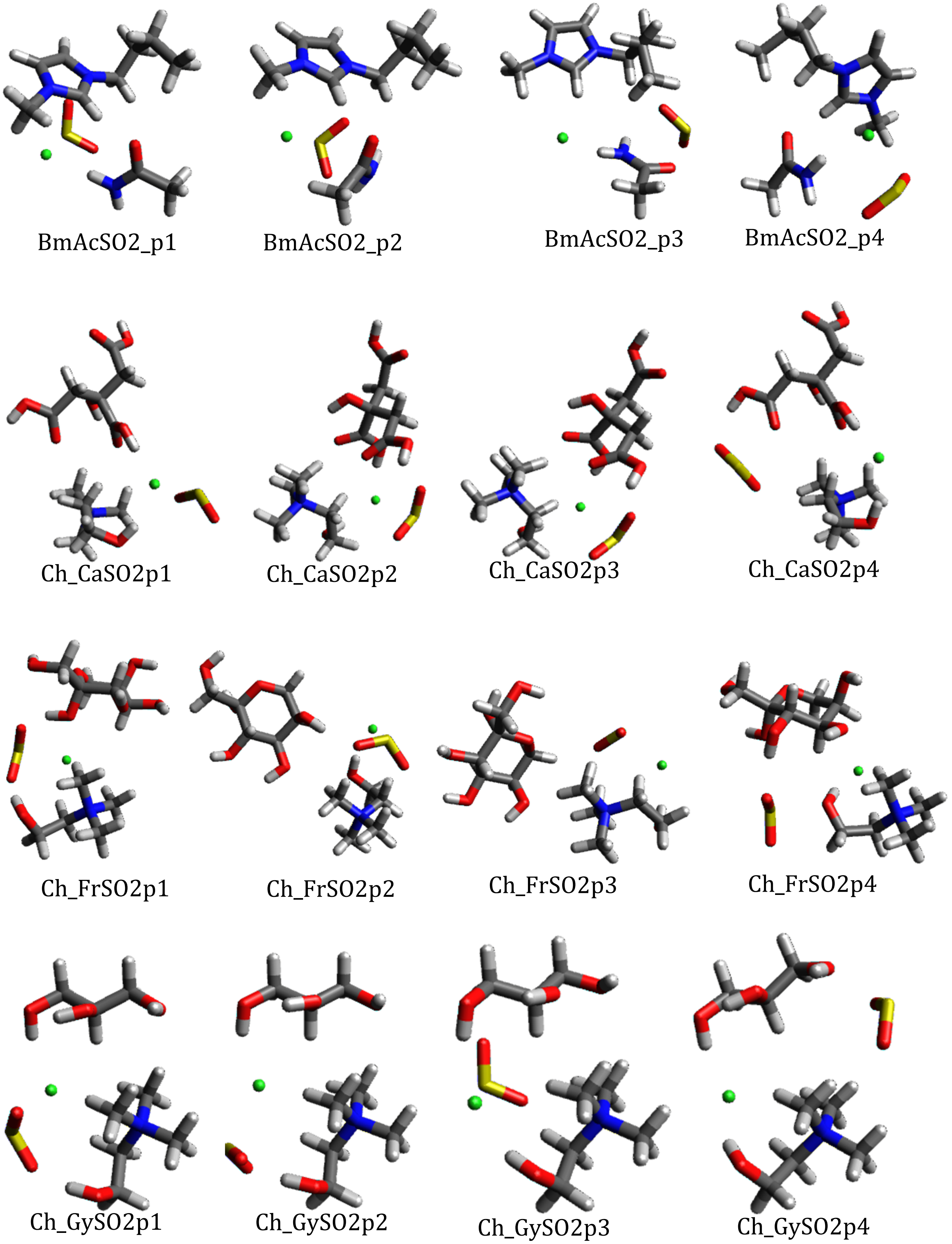

Figure S1. Finalized geometry optimizations for each studied DES+SO2 system at different spatial positions of SO2 around DES.

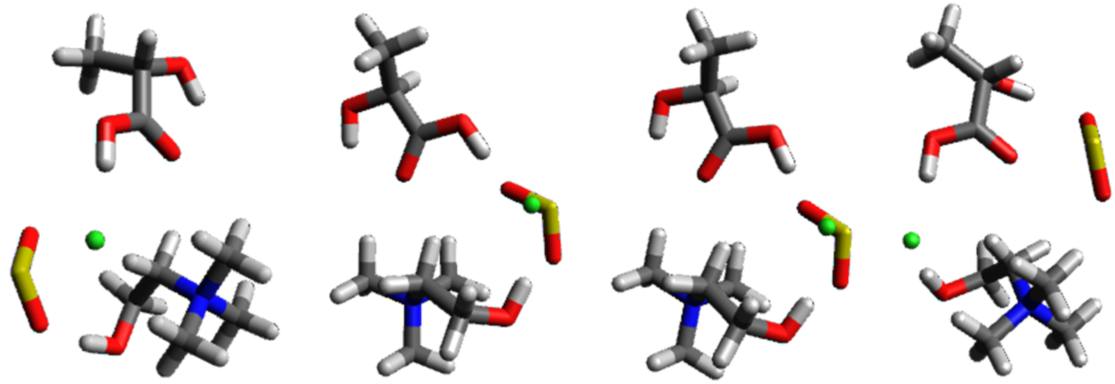

Ch_LaSO2p1 Ch_LaSO2p2 Ch_LaSO2p3 Ch_LaSO2p4

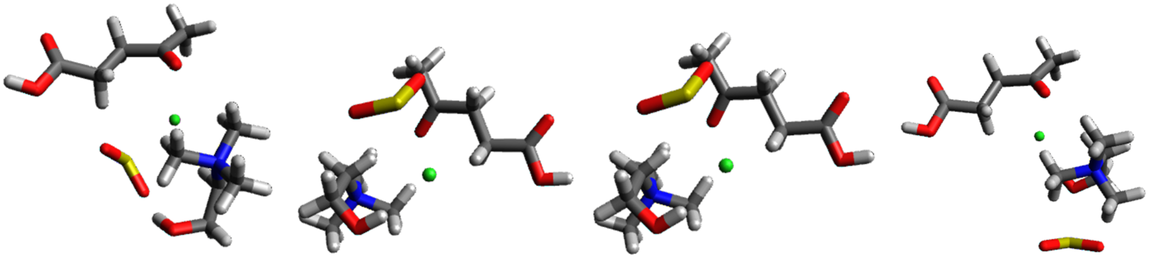

Ch_LvSO2p1 Ch_LvSO2p2 Ch_LvSO2p3 Ch_LvSO2p4

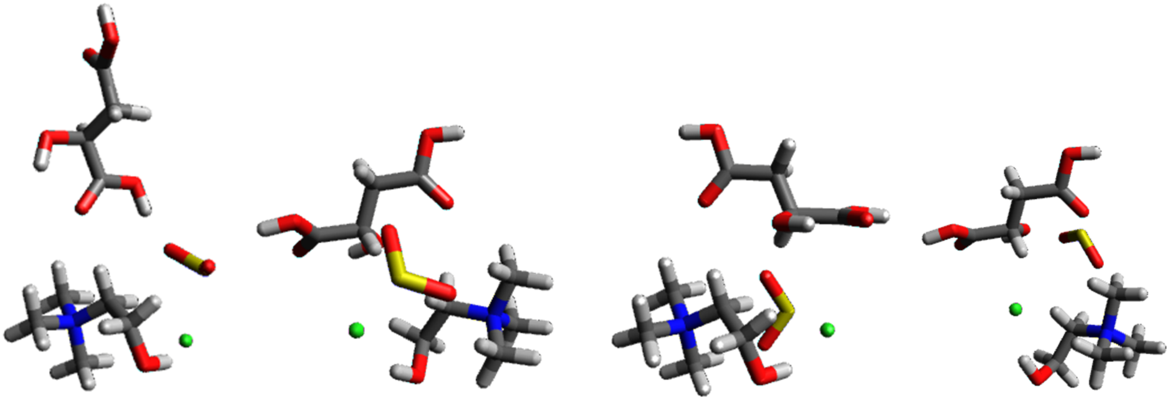

Ch_MaSO2p1 Ch_MaSO2p2 Ch_MaSO2p3 Ch_MaSO2p4

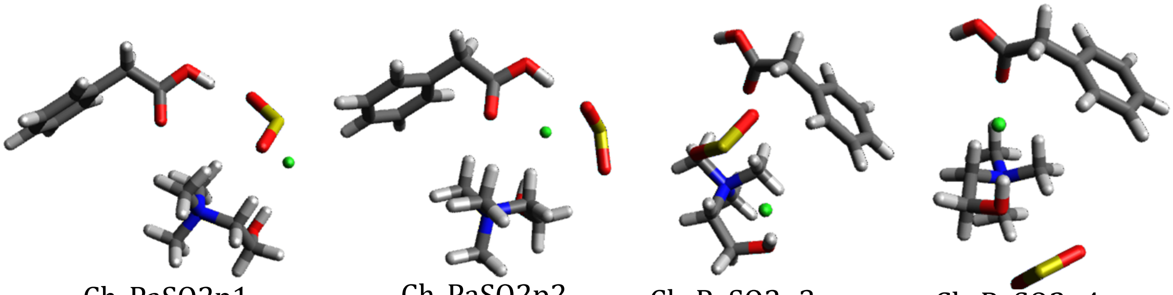

Ch_PaSO2p1 Ch_PaSO2p2 Ch_PaSO2p3 Ch_PaSO2p4

Figure S1. (con't)

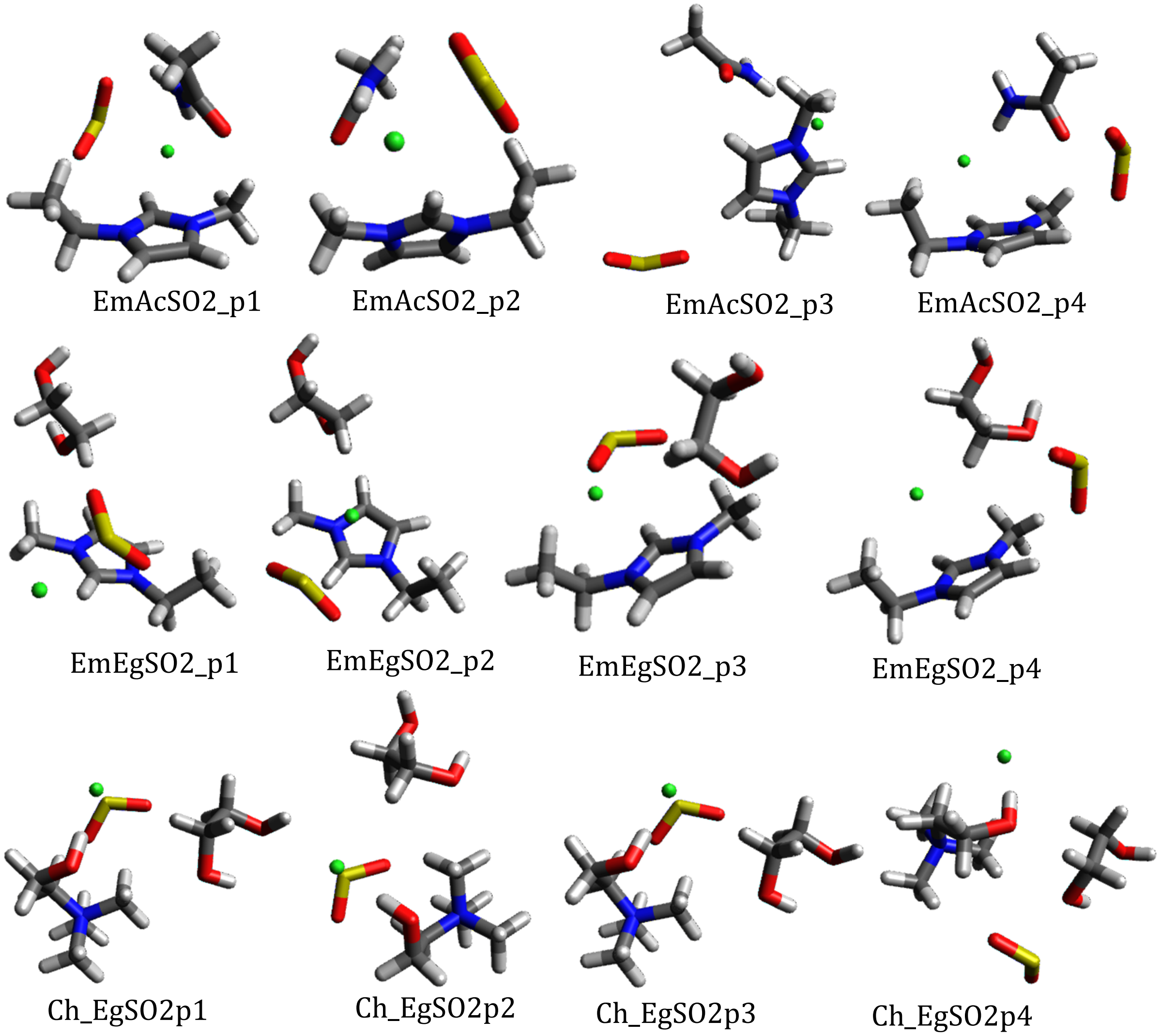

Figure S1. (con't)

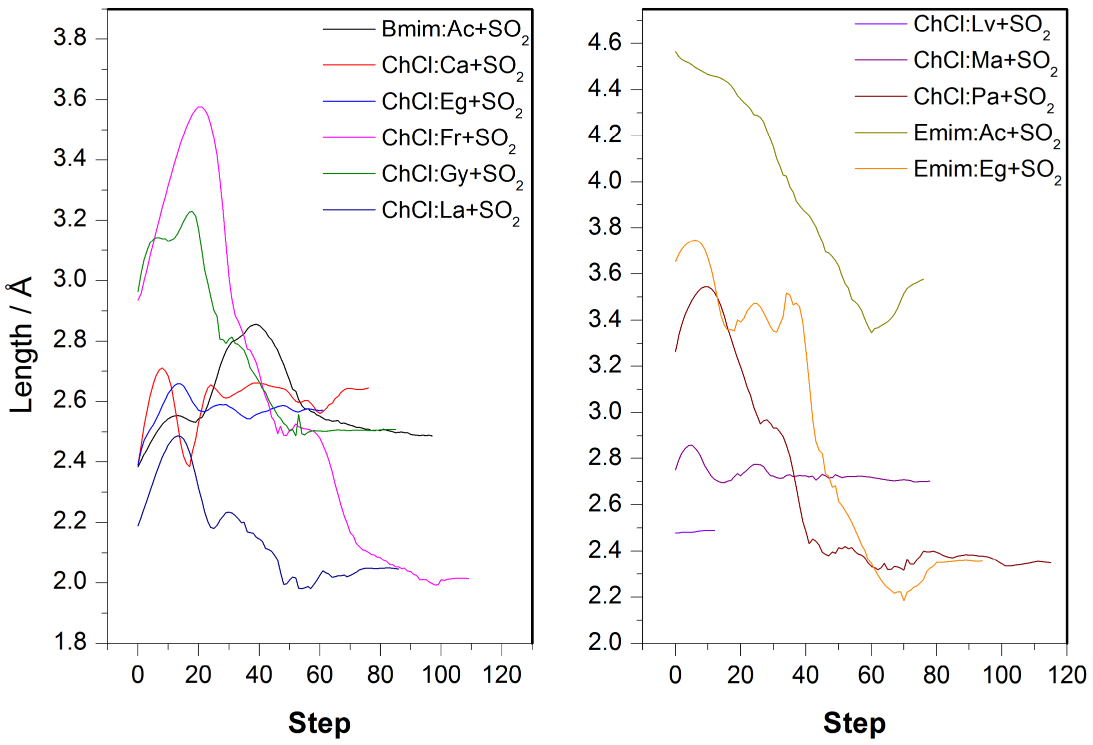

Figure S2. Distance evolution between BCP forming sites (DES···SO_2) throughout the geometry optimization.

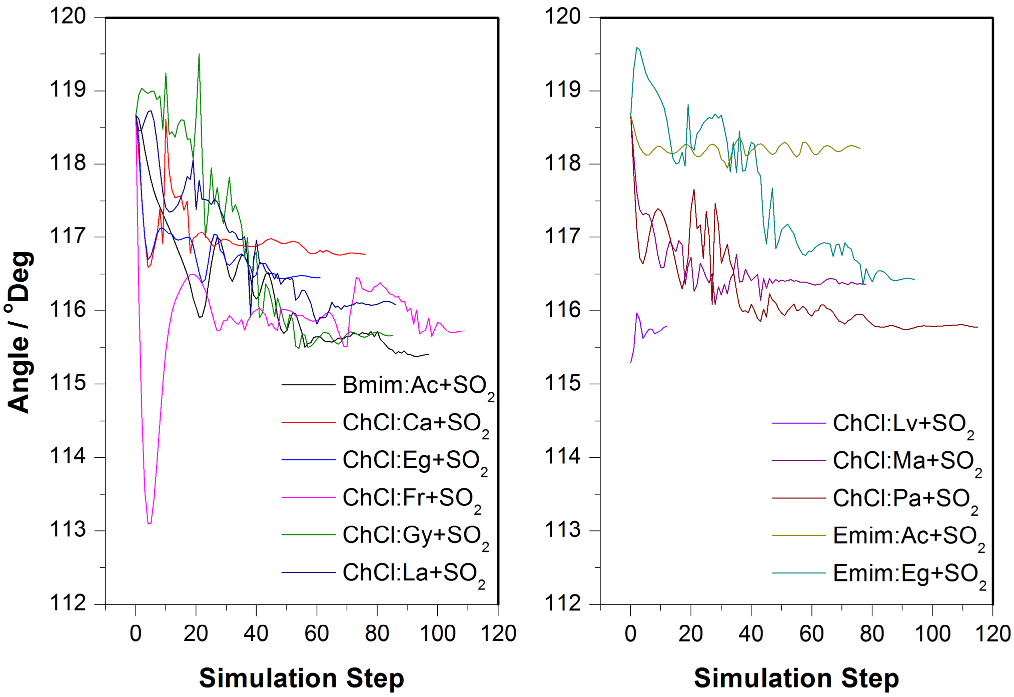

Figure S3. SO_2 angle evolution for each DES+SO_2 case throughout the geometry optimization.
